# Application of a circular-shaped pulsed field ablation catheter with magnetic sensors for pulmonary vein isolation: a multi-centre clinical study report

**DOI:** 10.1093/europace/euae068

**Published:** 2024-04-08

**Authors:** Yan Wang, Heng Li Lai, Qi Chen, Hao Liu, Qi Ming Liu, Wei Bin Huang, Yu Tao, Qingmei Xiong, Ning Zhou, Chunxia Zhao, Jie Qiu, Ziqin Xu, Dao Wen Wang

**Affiliations:** Division of Cardiology, Department of Internal Medicine, Tongji Hospital, Tongji Medical College, Huazhong University of Science and Technology, Jiefang Avenue 1095#, 430030 Wuhan, China; Division of Cardiology, Jiangxi Provincial People’s Hospital, Nanchang, China; Department of Cardiovascular Medicine, The Second Affiliated Hospital of Nanchang University, Nanchang, China; Division of Cardiology, The Second Affiliated Hospital of Guangxi Medical University, Nanning, China; Division of Cardiology, Xiamen University Affiliated Zhongshan Hospital, Xiamen, China; Division of Cardiology, The Second Xiangya Hospital of Central South University, Changsha, China; Division of Cardiology, Jiangxi Provincial People’s Hospital, Nanchang, China; Department of Cardiovascular Medicine, The Second Affiliated Hospital of Nanchang University, Nanchang, China; Division of Cardiology, Department of Internal Medicine, Tongji Hospital, Tongji Medical College, Huazhong University of Science and Technology, Jiefang Avenue 1095#, 430030 Wuhan, China; Division of Cardiology, Department of Internal Medicine, Tongji Hospital, Tongji Medical College, Huazhong University of Science and Technology, Jiefang Avenue 1095#, 430030 Wuhan, China; Division of Cardiology, Department of Internal Medicine, Tongji Hospital, Tongji Medical College, Huazhong University of Science and Technology, Jiefang Avenue 1095#, 430030 Wuhan, China; Division of Cardiology, Department of Internal Medicine, Tongji Hospital, Tongji Medical College, Huazhong University of Science and Technology, Jiefang Avenue 1095#, 430030 Wuhan, China; Division of Cardiology, Department of Internal Medicine, Tongji Hospital, Tongji Medical College, Huazhong University of Science and Technology, Jiefang Avenue 1095#, 430030 Wuhan, China

**Keywords:** Atrial fibrillation, Catheter ablation, Pulmonary vein isolation, Pulsed field ablation, Electroporation

## Abstract

**Aims:**

A few studies have reported the effect and safety of pulsed field ablation (PFA) catheters for ablating atrial fibrillation (AF), which were mainly based on basket-shaped or flower-shaped designs. However, the clinical application of a circular-shaped multi-electrode catheter with magnetic sensors is very limited. To study the efficacy and safety of a PFA system in patients with paroxysmal AF using a circular-shaped multi-electrode catheter equipped with magnetic sensors for pulmonary vein isolation (PVI).

**Methods and results:**

A novel proprietary bipolar PFA system was used for PVI, which utilized a circular-shaped multi-electrode catheter with magnetic sensors and allowed for three-dimensional model reconstruction, mapping, and ablation in one map. To evaluate the efficacy, efficiency, and safety of this PFA system, a prospective, multi-centre, single-armed, pre-market clinical study was performed. From July 2021 to December 2022, 151 patients with paroxysmal AF were included and underwent PVI. The study examined procedure time, immediate success rate, procedural success rate at 12 months, and relevant complications. In all 151 patients, all the pulmonary veins were acutely isolated using the studied system. Pulsed field ablation delivery was 78.4 ± 41.8 times and 31.3 ± 16.7 ms per patient. Skin-to-skin procedure time was 74.2 ± 29.8 min, and fluoroscopy time was 13.1 ± 7.6 min. The initial 11 (7.2%) cases underwent procedures with deep sedation anaesthesia, and the following cases underwent local anaesthesia. In the initial 11 cases, 4 cases (36.4%) presented transient vagal responses, and the rest were all successfully preventatively treated with atropine injection and rapid fluid infusion. No severe complications were found during or after the procedure. During follow-up, 3 cases experienced atrial flutter, and 11 cases had AF recurrence. The estimated 12-month Kaplan–Meier of freedom from arrhythmia was 88.4%.

**Conclusion:**

The PFA system, comprised of a circular PFA catheter with magnetic sensors, could rapidly achieve PVI under three-dimensional guidance and demonstrated excellent safety with comparable effects.

## Graphical Abstract

**Graphical Abstract:**
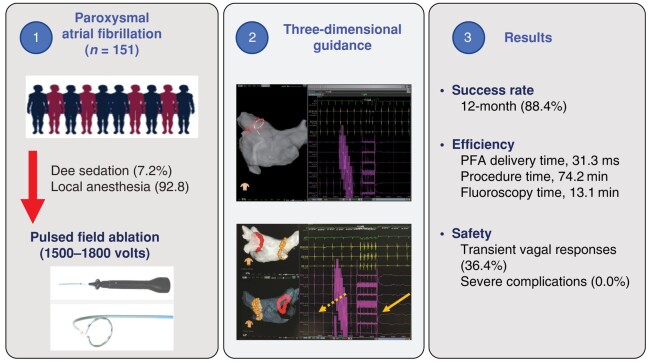

**What’s new?**

- A novel proprietary bipolar pulsed field ablation (PFA) system was used for pulmonary vein isolation (PVI), which utilized a circular-shaped multi-electrode catheter with magnetic sensors and allowed for three-dimensional model reconstruction, mapping, and ablation in one map.
- This PFA system can achieve rapid PVI under three-dimensional guidance with excellent safety, comparable effects, and good tolerance in local anaesthesia.

## Introduction

Atrial fibrillation (AF) is a common arrhythmia, with patients often experiencing palpitations, and may lead to systemic embolism such as stroke or limb embolism, loss of heart function, and significantly worsen the existing heart failure.^1^ Currently, catheter ablation has become the most important radical treatment method for AF. Traditional methods use radiofrequency catheter ablation. However, radiofrequency heating may damage the surrounding organs of the heart, generate char, thrombosis, and bubbles, and result in heart tissue burst, which will increase the risk of cardiac tamponade.^2^

Pulsed field ablation (PFA), a novel ‘non-thermal’ ablation modality recently applied for pulmonary vein isolation (PVI) in patients with AF, has shown great advantages regarding safety and efficacy over traditional radiofrequency ablation.^3^ So far, a few studies have reported the use of PFA catheters, which were based on basket-shaped or flower-shaped designs for PVI.^4^ However, limited data reported the clinical application of a circular-shaped multi-electrode catheter with magnetic sensors, especially power delivered with three-dimensional navigation.

Here, we report the application of a novel PFA system utilizing a circular-shaped multi-electrode catheter with magnetic sensors for model reconstruction, mapping, and pulmonary isolation in one three-dimensional map in patients with paroxysmal AF.

## Methods

### Trial design

ESPFA-CN21 (https://www.clinicaltrials.gov; unique identifier: NCT05400928) is a prospective, multi-centre, single-armed, pre-market clinical trial to evaluate the efficacy, efficiency, and safety of a novel PFA system for PVI in patients with paroxysmal AF. LEAD-PFA (Sichuan Jinjiang Electronic Medical Device Technology Co., Ltd, Chengdu, China), a proprietary bipolar PFA system, has been designed for PVI. The system utilizes a circular-shaped PFA catheter embedded with three magnetic sensors allowing model reconstruction, mapping, and ablation in one map with three-dimensional navigation. The distal end is 5.5 Fr and the proximal end is 8 Fr in diameter, which can be used for ablation via a routinely used 8.5 Fr Swartz sheath. This trial was approved by the China National Drug Administration and the institutional ethics review board of Tongji Hospital, Tongji Medical College, Huazhong University of Science and Technology, according to the guidelines for good clinical practice and the Declaration of Helsinki.

### Patient selection and data collection

Patients with paroxysmal AF were enrolled if they planned to undergo ablation procedures and agreed to participate in this trial. The patients were excluded if they presented with a prior history of ablation for AF, a left ventricular ejection fraction <35%, or a left atrial diameter (LAD) >55 mm. The combined morbidity of atrial flutter was not excluded, which was planned to be ablated with the PFA method first or with the traditional radiofrequency method if the PFA method failed.

The study examined fluoroscopic time, procedure time, immediate success rate, procedural success rate at 12 months after the procedure, and relevant procedural complications. Procedure time is the amount of time taken from the start of the skin puncture for venous access to the removal of all catheters. Immediate success is defined as the achievement of complete electrical isolation of all the pulmonary veins, which is shown as an afferent and efferent conduction block. The procedural success rate at 12 months after the procedure was defined as the absence of AF, atrial flutter, or atrial tachycardia (duration ≥30 s) without the use of anti-arrhythmic drugs between the 3rd and 12th months after ablation. Fluoroscopy time (in minutes) was defined as the total duration of exposure during the procedure. The total ablation time was calculated in milliseconds.

Independent researchers recorded and analysed the possible complications, including pseudo-aneurysm, arterial-venous fistula, newly occurred atrioventricular block, cardiac tamponade, adjacent nerve injury (e.g. phrenic nerve injury), pneumothorax, haemothorax, lung injury, pulmonary vein stenosis or severe gastroparesis, oesophageal injury, embolisms such as stroke, or any other complication that required further intervention suspected to be related to ablation.

Other independent researchers collected and recorded all pre-operative, operative, and follow-up data. The data included clinical and demographic variables, procedure-related variables (procedure date, puncture route, ablation method, procedure time, fluoroscopy time, number of lesions, total ablation time, immediate success rate, and complications), medications, and recurrences during follow-up.

### Ablation procedure

Anti-arrhythmic medications were discontinued for at least five half-lives before ablation. Amiodarone was discontinued 6 months before the procedure. Deep anaesthetic sedation or local anaesthesia was used. Laryngeal mask ventilation or tracheal intubation was chosen according to the patient’s condition.

Written informed consents for participating in the clinical trial and for catheter ablation were obtained. After anaesthesia, a steerable coronary sinus electrode was placed through an 8 Fr short sheath via the right femoral vein. An 8.5 Fr Swartz SL1 transseptal sheath with dilator was also introduced from the right femoral vein. A transseptal puncture was performed under fluoroscopic guidance and/or with transoesophageal echocardiography. Heparin 80–100 U/kg was administered after a successful transseptal puncture. Activated clotting time was measured and adjusted to maintain between 250 and 350 s.

A circular PFA catheter with magnetic sensors was used to establish the model, usually for the left atrium and pulmonary veins. The left and right pulmonary antra were labelled. For the convenience of observation, the models were copied and presented in a grid-perspective mode. Using PFA, the isolation of the left pulmonary vein was labelled on one model, and the right pulmonary vein was on another.

The voltage output can be divided into five levels, namely 500–800, 800–1000, 1000–1300, 1300–1500, and 1500–1800 V. The voltage used for PVI is 1500–1800 V.

The following evidence indicated a successful PVI: (i) pulmonary vein potential disappeared or was disassociated; (ii) voltage mapping confirmed PVI; (iii) pacing could not capture the adjacent atrium; the pacing was performed at multiple sites by the PFA catheter along the antrum within the ablation line; a triple threshold value was used, and the distance between two adjacent pacing sites was <1 cm; and (iv) after intravenous infusion of isoproterenol, there was no pulmonary vein potential detected within the ablation line again, and pacing within the ablation line could not capture the atrium.^5^

### Follow-up

Independent researchers conducted follow-ups 10 days, 1, 3, 6, 12, and 15 months following the operation. During follow-up, every patient typically took an electrocardiogram (ECG), echocardiogram, and physical examination. Twenty-four hours Holter was scheduled to be regularly conducted at the screening stage prior to enrolment, in the 6th and 12th month after the procedure. Any symptoms, signs, or medication were recorded if they were suspected to be related to complications or recurrence. When a patient exhibits suspicious symptoms or signs, an ECG and Holter would be conducted to rule out a recurrence.

### Statistical analysis

All continuous data are presented as mean ± standard deviation, whereas categorical data are expressed as counts and percentages. Student’s *t*-tests, one-way analysis of variance, χ^2^ tests, and Fisher’s exact tests were used to compare differences among the groups. All analyses were performed using the.Statistical Package for the Social Sciences version 13.0 (IBM Inc., Armonk, NY, USA). All *P*-values <0.05 were considered statistically significant. Kaplan–Meier event-free survival analysis was conducted to assess the cumulative freedom from recurrence.

## Results

### Study population

Between July 2021 and December 2022, 151 consecutive patients from 7 centres with paroxysmal AF who agreed to participate in this clinical trial were enrolled. Eighty-nine (58.9%) of them were male. The average LAD was 36.8 ± 6.2 mm. The baseline characteristics of the enrolled patients are shown in *Table 1*.

**Table 1:** Baseline characteristics of the enrolled patients

Variable	Result
With AT/AF, *n* (%)	8 (5.3)
Age (years)	59.0 ± 10.3
Weight (kg)	67.1 ± 11.1
Height (cm)	164.0 ± 7.8
BMI	24.9 ± 3.5
Patients with risk factors, *n* (%)	
Severe COPD	0
CAD	21(13.9)
Hypertension	63(41.7)
Diabetes	15(9.9)
Prior stroke/TIA	4(2.7)
History of hyperthyroidism	4(2.7)
Hypothyroidism	7(4.6)
Smoking history	13(8.6)
LA (mm)	36.81(6.2)
LA diameter >40 mm, *n* (%)	41(27.2)
LA diameter >45 mm, *n* (%)	14(8.6)
Moderate–severe valvular regurgitation	0
LV (mm)	48.5 ± 4.1
LVEF (%)	62.8 ± 6.3
NYHA	
I, *n* (%)	88(58.28)
II, *n* (%)	22(14.57)

AT, atrial tachycardia; AF, atrial flutter; SD, standard deviation; BMI, body mass index; COPD, chronic obstructive pulmonary disease; CAD, coronary artery disease; TIA, transient ischemic attack; LA, left atrium; LV, left ventricle; LVEF, left ventricular ejection fraction; NYHA, New York Heart Association.

### Procedural characteristics

**Figure 1:**
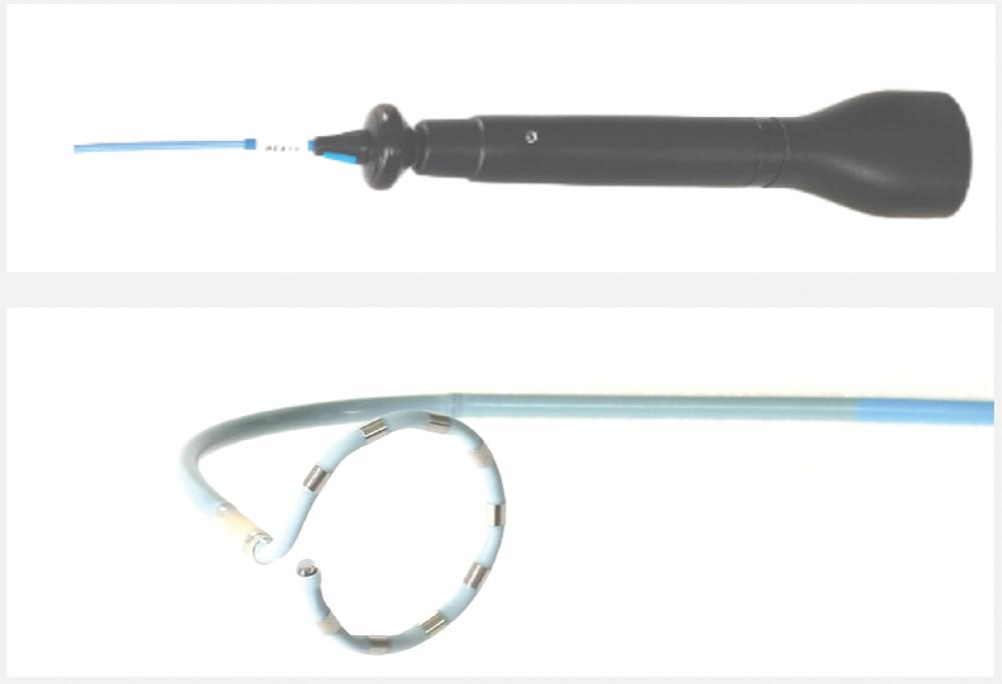
Pulsed field ablation catheter, a circular-shaped catheter embedded with magnetic sensors at the tip, allows mapping, model reconstruction, and ablation. The distal end was 5.5 Fr and the proximal end was 8 Fr in diameter, which could be used for ablation via routinely used 8.5 Fr Swartz sheath.

A circular-shaped multi-electrode catheter with magnetic sensors was used for model reconstruction, mapping, and PFA. The catheter could apply ablation from all electrodes or some selected pairs of adjacent electrodes according to the operator’s needs (*Figure 1*).

**Figure 2:**
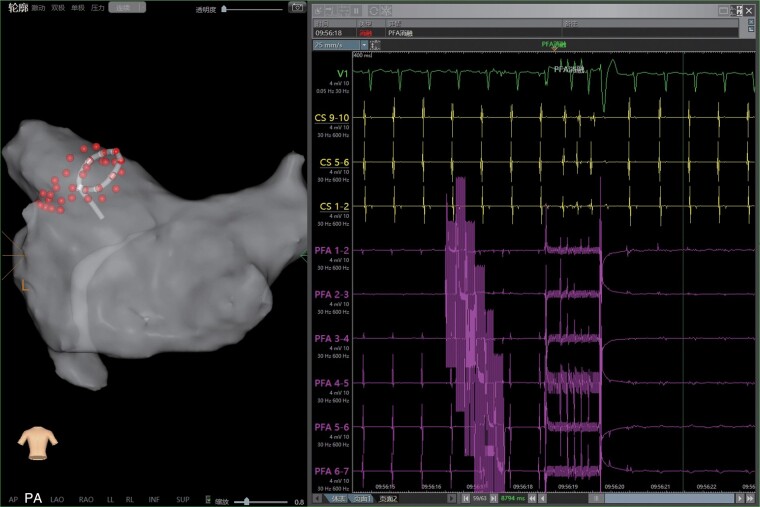
The PFA was delivered at the anterosuperior aspect of the left antrum for isolation of the LSPV, which was shown on the left panel in posterior–anterior view. The dots labeled near the ostium of left pulmonary vein indicated the location of lesions formed during catheter ablation. The upper part of the right panel showed an intra-cardiac electrogram recorded from the coronary sinus electrode. The lower part of the right panel showed the immediate loss of atrial potential after the delivery of PFA, which was recorded from the PFA catheter. LSPV, left superior pulmonary vein; PFA, pulsed field ablation.

**Figure 3:**
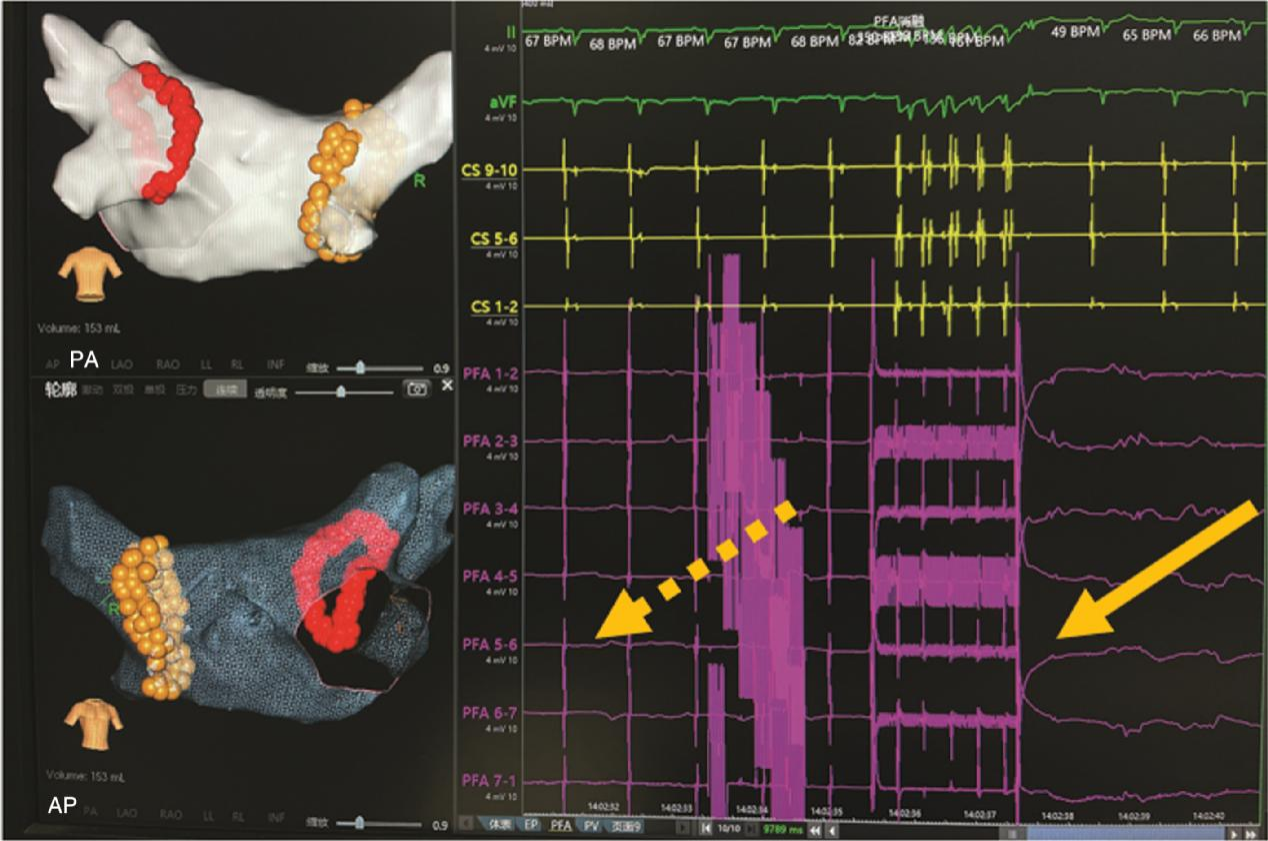
The PFA was delivered at the anterior aspect of the right antrum for the isolation of RSPV, which was shown on the left panel in the posterior–anterior view. Please note that for the convenience and visual display of the outer edge of the injury, the technician has intentionally removed the lesion points inside the ablated antrum line in this figure. The actual distribution of the lesions was scattered, as shown in the above figure. The electrogram in yellow on the right panel showed an intra-cardiac electrogram recorded from the coronary sinus electrode. The arrows on the right panel showed the immediate loss of atrial potential after the delivery of PFA, which was recorded from the PFA catheter. PFA, pulsed field ablation; RSPV, right superior pulmonary vein.

**Figure 4:**
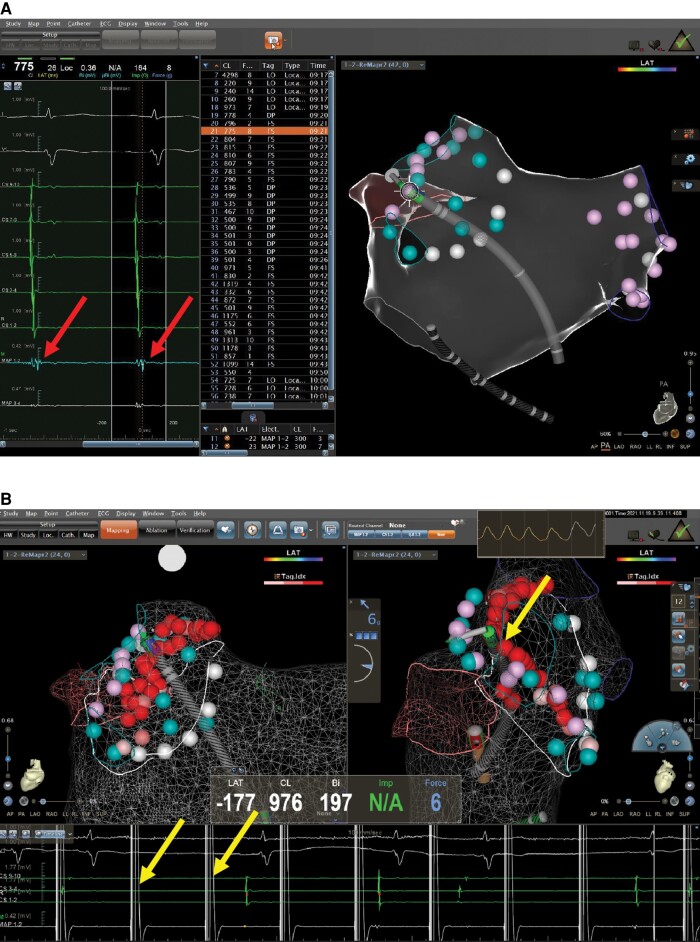
A redo patient, who had a recurrence of atrial fibrillation in the second month and still had a recurrence in the sixth month after PFA, was ablated using the traditional radiofrequency method because of frequent and severe palpitations. However, he had a recurrence soon after radiofrequency ablation and was ablated for the third time using the radiofrequency method. He had no recurrence during the recent follow-up of 14 months. Large atrial potentials could be recorded from the radiofrequency ablation catheter, which was placed within the left antrum; the locations of the catheter were shown on the right side of the upper panel in a posterior–anterior view. The electrogram on the lower panel confirmed the isolation of the left antrum by pacing within the antrum with a radiofrequency ablation catheter. The radiofrequency ablation catheter was ThermoCool SmartTouch® SF (Biosense Webster Inc., Diamond Bar, CA, USA). PFA, pulsed field ablation.

In all 151 patients, all pulmonary veins were acutely isolated by biphasic PFA (*Figures 2* and *3*). Pulsed field ablation delivery time per patient was 31.3 ± 16.7 ms, skin-to-skin procedure time was 74.2 ± 29.8 min, and fluoroscopy time was 13.1 ± 7.6 min (*Table 2*). The initial 11 (7.2%) cases underwent deep sedation anaesthesia using laryngeal mask ventilation, and all the rest underwent local anaesthesia. Also, in these five cases, oesophageal ultrasound was used for monitoring, and only microbubbles occasionally could be seen during ablation. Eight cases underwent a redo procedure using the radiofrequency ablation method during the follow-up period (*Figure 4*).

**Table 2:** Procedure outcomes

Variable	Result
Procedure time (min)	74.2 ± 29.8
Fluoroscopy time (min)	13.1 ± 7.6
Acute isolation of pulmonary vein	151 (100%)
PFA delivery (times)	78.4 ± 41.8
LPV (times)	40.5 ± 20.1
RPV (times)	37.9 ± 23.2
PFA delivery time (ms)	31.3 ± 16.7
Average length of stay (days)	6.6 ± 3.3
AT recurrence	0
AF recurrence	3 (2.0%)
AF recurrence	11 (7.3%)

PFA, pulsed field ablation; LPV, left pulmonary vein; RPV, right pulmonary vein; other abbreviations were shown as in Table 1.

### Combined arrhythmias

Eight (5.3%) patients had combined an atrial flutter or focal atrial tachycardia. Among them, five patients had been diagnosed with concomitant atrial flutter before the enrolment, two patients experienced atrial flutter during the electrophysiology study, and one patient experienced two kinds of focal atrial tachycardia during the procedure. The atrial flutter was ablated with a radiofrequency ablation catheter. The focal atrial tachycardia was mapped and ablated with the PFA catheter by the selected local pair of electrodes.

### Final success rate

The average time of index recurrence after PFA was 6 months. During follow-up, 2 cases experienced atrial flutter, and 24 cases had a recurrence of AF. The estimated 12-month Kaplan–Meier of freedom from AF was 88.4%.

### Complications

In the initial 11 ablation patients, 4 cases (36.4%) presented a transient intense vagal response when the anterosuperior wall of the left superior pulmonary vein (LSPV) was ablated, which had a significant blood pressure decrease (median 32 mmHg) and a heart rate decrease (median 43 b.p.m.). During the following study, the vagal responses were all preventatively treated with atropine injection and fluid infusion. No complications requiring special treatment were observed during or after the procedure. There was one pseudo-aneurysm, which was well treated with elastic bandage compression and discharged 2 weeks later.

One patient had a cerebellar haemorrhage, and one patient had an acute non–ST-elevation acute myocardial infarction in the 11 and 10 months after PFA, respectively, which were considered to have no association with PFA.

Beyond the above complications, no patient presented symptoms or signs suggestive of additional primary adverse events during follow-up, including transient ischemic attack (TIA) or stroke, visceral or limb embolism, phrenic nerve injury, pulmonary vein stenosis, and oesophageal injury.

## Discussion

Pulmonary vein isolation has been an important step in the ablation of AF, especially paroxysmal AF. As mentioned in Boersma’s review, the past 25 years have seen an incredible surge in scientific interest in developing new catheters for reliable and efficient PVI. As we know, radiofrequency catheters and cryoballoons have been used as the primary tools for PVI in the past. In recent years, PFA, a newly emerging method, has been introduced for PVI, showing encouraging prospects.^6^

As for catheter ablation methods of AF, compared with the traditional radiofrequency method, PFA has the following primary advantages: (i) it is relatively tissue-specific; using a certain pulsed field intensity, the pulse energy can specifically damage myocardial cells, but not smooth muscle cells, endothelial cells, or neural cells, thus reducing the related complications such as oesophageal injury, phrenic nerve paralysis, and pulmonary vein stenosis. (ii) PFA can apply ablation much more efficient since it can deliver power from multiple electrodes simultaneously, and the time of each ablation is reduced from tens of seconds to <1 s (usually at a millisecond or nanosecond level). (iii) The damage of PFA relies on the perforation of cell membranes rather than thermal damage, thus greatly reducing the potential risk of char formation, tissue pop, and excessive heating of adjacent tissue.^7^

However, as a novel technique, PFA still has many issues with which to be concerned.^7^ How to design a catheter to isolate the pulmonary veins efficiently? What parameter package can cause durable damage? How to integrate it with a three-dimensional navigation system?

Catheters shaped like baskets or flowers have been used in most previous reports for PFA, which may decrease the number of manipulations and ablation times. However, the catheter is relatively larger, and the operation may require more caution and gentleness. Also, a few studies reported the use of circular-shaped PFA catheters for AF ablation, including Pulse Select from Medtronic and the InspIRE study from Abbott, which all showed a satisfactory success rate. In addition, a recent report showed that the application of an expandable lattice catheter might have some advantages.^8–10^

Here, we explored the effect of a circular ablation catheter with magnetic sensors using different parameters of packet for PVI.

### Safety

Regarding the risk of pulmonary vein stenosis, it has been reported that an overall incidence rate is 36.4% and a moderate-to-severe incidence rate of ∼5% when the traditional radiofrequency method is used for AF ablation, whereas the overall incidence is <1%, and there is no moderate-to-severe stenosis with the PFA method.^11,12^ Although we did not routinely perform pulmonary vein computer tomography during follow-up, none of our studied cases experienced symptoms of pulmonary vein stenosis. In addition, eight patients underwent redo ablation due to a recurrence during follow-up, and the pulmonary vein reconstruction in those patients showed no stenosis.

Oesophagus damage is also a concern when AF ablation is performed, especially during left atrial ablation on the posterior wall. It had been reported that ∼25% had symptoms related to oesophageal injury and 0.2% had symptoms related to gastroparesis after traditional radiofrequency ablation.^13,14^ Unlike radiofrequency ablation, PFA seems to avoid irreparable oesophageal damage. Studies have found that the oesophageal lesions caused by PFA were restricted to the muscle layer within the luminal epithelial layer.^15,16^ Consistent with the MANIFEST-PF trials,^17^ none of the patients in our study revealed related symptoms of obvious oesophageal injury or gastric paresis after PFA.

Another concern about PFA is its effect on the nervous system. Experimental and numerical simulation sources have demonstrated nerve resilience to PFA, where the nerves are unaffected, are recovered, or demonstrate preserved endoneural architecture.^18,19^ This may be attributed to the insulating effects of the Schwann cell sheath or the smaller diameter of axons, resulting in the relatively minimal generation of an electromotive force during the application of high voltage fields. It is worth noting that a relatively high incidence of intense vagal response was found in our work when the anterosuperior aspect of the LSPV was ablated with PFA. In the initial dozen cases, ∼23% of patients experienced an obvious decrease in blood pressure and heart rate (∼30–40 b.p.m.). In the subsequent cases, this response was prevented by the preventative use of atropine and rapid fluid infusion. We considered that the intense vagal response was caused by high-frequency stimulation rather than by nerve injury.

As we know, bubbles may be generated by inappropriately intense PFA, which may increase the risk of systemic embolism. For the sake of safety, we attempted intensity-step-wise ablation in the initial stage. Oesophageal ultrasound monitoring revealed that only microbubbles could sometimes be seen even using the strongest designed energy, which was 1800 V in this trial. Those microbubbles quickly dissolved before leaving the left atrium. Except for the complications mentioned above, there were no manifestations of pericardial tamponade, stroke, organ and limb thrombosis, phrenic nerve injury, or lung injury.

### Efficacy

It has been reported that the 1-year success rate has been ∼73.3–84.5% when PFA is used for PVI in patients with paroxysmal AF^4,20,21^ and ∼71.3–86.6% when the traditional radiofrequency method is used.^20,22^ Recently, the EU-PORIA registry, a real-world PFA study, has recently shown that the success rate of paroxysmal AF exceeds 80% in 1 year.^23^

The studied system also achieved a satisfactory success rate. The 1-year success rate of this trial was ∼88.4%. Our success rate differs from those of other studies using circular catheters, such as the PULSED AF study or the InspIRE study, possibly due to different follow-up frequencies and different enrolled populations or partially due to our more aggressive ablation strategy. Our ablation time for each pulmonary vein was almost twice that of those studies, and we even ablated a little deeper within the pulmonary antrum.^8,9^

Also, the PFA system is efficient and only requires a very short learning curve. Using the designed circular catheter, we could perform model reconstruction, voltage mapping, and the PFA procedures, which were all applied under three-dimensional guidance. In addition, the circular PFA catheter is 8 Fr in diameter, and the operation can be performed through a routine atrial septal puncture sheath. The average fluoroscopy time was about 13 min, and the procedure time was about 74.2 min.

As for anaesthesia, over 80% of patients in the PULSED AF study received general anaesthesia, and over 70% of patients in the inspIRE study used general anaesthesia.^8,9^

Using this designed package of biphasic waves, all the patients underwent the procedures with good tolerance, which revealed no obvious pain or muscle contraction. Although a few patients showed slight to moderate diaphragmatic twitching when the PFA was applied at the anterosuperior aspect of right superior pulmonary vein (RSPV), which led to phrenic nerve stimulation, in this situation, we usually reduced the number of ablation electrodes to attenuate the amplitude of diaphragmatic twitching. Anyway, the position of patients did not change, and reconstruction of the atrial model was unnecessary.

At first, we were not sure whether the patients could tolerate the designed PFA ablation energy in a conscious state. Thus, we performed PFA using deep sedation in the initial 11 cases. However, we found no muscle twitching phenomenon during ablation; then, we intentionally attempted several times of enhanced pulse ablation in the pulmonary antrum after the patient regained consciousness, and the patients were all well-tolerated. Hence, we changed to local anaesthesia in the subsequent procedure.

### Outlook

Unlike radiofrequency ablation, PFA can damage local tissues even without contact. However, some data have shown that non-contact with the PFA catheter can significantly reduce its effect. In the initial 10 cases, we simultaneously delivered a pulse with all of the electrodes, while in the later cases, we chose electrodes that were expected to contact well with the target tissue (such as the top electrodes in the direction of the flexed catheter) to avoid ineffective ablation. Therefore, to monitor whether the electrodes are fully attached, future PFA catheters need to add pressure sensors or use a tissue proximity index.

Although the use of our circularly designed PFA catheter possesses many advantages mentioned above, it has the following shortcomings: (i) it is difficult to stably adhere to certain positions, such as the top of the RSPV and the left Warfarin ridge; to overcome the above issues, we withdrew some proximal electrodes into the introducing sheath (SL1 sheath), resulting in a C-shaped bending of the distal electrodes, which can enhance the catheter stability at the top of the RSPV; of course, if a steerable sheath is used, the above issues may be improved to some extent. (ii) When a patient has atrial flutter, an annular catheter makes it difficult to effectively ablate the uneven tricuspid isthmus.

Therefore, future PFA catheters, when combined with straight-ended catheters and with the capability of pressure monitoring, may significantly overcome the aforementioned disadvantages.

## Conclusions

Our designed circular PFA system with magnetic sensors can achieve rapid PVI under three-dimensional guidance with excellent safety and comparable effects. It may be more efficient if a straight-ended catheter with the capability of pressure monitoring is combined.

## Supplementary Material

euae068_Supplementary_Data

## Data Availability

All relevant data are within the manuscript and its Supplementary material.
